# Differential Gene Expression in Soybean Leaf Tissues at Late Developmental Stages under Drought Stress Revealed by Genome-Wide Transcriptome Analysis

**DOI:** 10.1371/journal.pone.0049522

**Published:** 2012-11-19

**Authors:** Dung Tien Le, Rie Nishiyama, Yasuko Watanabe, Maho Tanaka, Motoaki Seki, Le Huy Ham, Kazuko Yamaguchi-Shinozaki, Kazuo Shinozaki, Lam-Son Phan Tran

**Affiliations:** 1 Signaling Pathway Research Unit, RIKEN Plant Science Center, Yokohama, Kanagawa, Japan; 2 National Key Laboratory of Plant Cell Biotechnology and Agricultural Genetics Institute, Vietnamese Academy of Agricultural Science, Hanoi, Vietnam; 3 Plant Genomic Network Research Team, RIKEN Plant Science Center, Yokohama, Kanagawa, Japan; 4 Japan International Research Center for Agricultural Sciences, Tsukuba, Ibaraki, Japan; 5 Gene Discovery Research Group, RIKEN Plant Science Center, Yokohama, Kanagawa, Japan; Michigan State University, United States of America

## Abstract

The availability of complete genome sequence of soybean has allowed research community to design the 66 K Affymetrix Soybean Array GeneChip for genome-wide expression profiling of soybean. In this study, we carried out microarray analysis of leaf tissues of soybean plants, which were subjected to drought stress from late vegetative V6 and from full bloom reproductive R2 stages. Our data analyses showed that out of 46093 soybean genes, which were predicted with high confidence among approximately 66000 putative genes, 41059 genes could be assigned with a known function. Using the criteria of a ratio change > = 2 and a q-value<0.05, we identified 1458 and 1818 upregulated and 1582 and 1688 downregulated genes in drought-stressed V6 and R2 leaves, respectively. These datasets were classified into 19 most abundant biological categories with similar proportions. There were only 612 and 463 genes that were overlapped among the upregulated and downregulated genes, respectively, in both stages, suggesting that both conserved and unconserved pathways might be involved in regulation of drought response in different stages of plant development. A comparative expression analysis using our datasets and that of drought stressed *Arabidopsis* leaves revealed the existence of both conserved and species-specific mechanisms that regulate drought responses. Many upregulated genes encode either regulatory proteins, such as transcription factors, including those with high homology to *Arabidopsis* DREB, NAC, AREB and ZAT/STZ transcription factors, kinases and two-component system members, or functional proteins, e.g. late embryogenesis-abundant proteins, glycosyltransferases, glycoside hydrolases, defensins and glyoxalase I family proteins. A detailed analysis of the *GmNAC* family and the hormone-related gene category showed that expression of many *GmNAC* and hormone-related genes was altered by drought in V6 and/or R2 leaves. Additionally, the downregulation of many photosynthesis-related genes, which contribute to growth retardation under drought stress, may serve as an adaptive mechanism for plant survival. This study has identified excellent drought-responsive candidate genes for in-depth characterization and future development of improved drought-tolerant transgenic soybeans.

## Introduction

Cultivated soybean (*Glycine max* L.) has been known as one of the major legume crops in the world, providing an abundant source of oil and protein-rich food for both human and animal consumption. The growth and productivity of soybean are adversely affected by various environmental stresses, among which drought stress is considered the harshest, affecting all stages of plant growth and development. Drought stress, which especially occurs at late vegetative stages, may cause significant yield losses, up to 40% in the bad year, and a reduction of seed quality for soybean [1]–[4].

In response to drought stress, plants, including soybean, activate a wide range of defense mechanisms that function to increase tolerance to water limiting conditions. The early events of plant responses to drought stress are the stress signal perception and subsequent signal transduction which lead to the activation of various molecular, biochemical and physiological responses [5]–[11]. With the availability of genomic sequences from various plant species and recent advances in microarray technologies, genes associated with drought/dehydration responses have been identified in a number of plant species, including both model plants, such as *Arabidopsis* [12], and crops, such as rice (*Oryza sativa*) [13], [14]. However, despite that the soybean genomic sequence was completed several years ago [15], and subsequently the 66 K Affymetrix soybean array platform, which covers all of the soybean genes annotated by the Glyma1 model, was designed by a US consortium, comprehensive genome-wide analysis of the soybean transcriptome under drought stress remained to be determined.

Keeping all these in mind, in this study we have performed a microarray analysis using the 66 K Affymetrix soybean GeneChip to gain an overall picture of transcriptome-wide changes in soybean leaves under drought stress. In this study, we imposed drought stress on soybean plants from late vegetative stage (V6) till early bloom reproductive stage (R1) and during full bloom R2 stage, and examined differential gene expression in V6 and R2 leaves of soybean plants grown under well-watered and drought conditions. The period from late V6 stage toward the end of R2 stage is known as one of critical periods that hurts yield (http://www.okstate.edu/OSU_Ag/oces/timely/soybean.htm), giving rise to the need of studying mechanisms of soybean responses to drought stress during this period aimed at developing drought-tolerant transgenic soybeans. Since the expression profiles of many genes obtained by qRT-PCR and microarray analysis were in good accordance, this array platform was found to be suitable for a high-throughput genome-wide analysis. Furthermore, the microarray data showed transcriptional changes of various well-known functional and regulatory genes; including transcription factors (TFs), kinases, heat shock proteins, late embryogenesis-abundant (LEA) proteins, osmoprotectant biosynthesis-related proteins, hormone-related proteins, transporters and detoxification enzymes. In addition, we have performed a comparative expression analysis of V6 and R2 microarray datasets to search for the conserved and unconserved sets of genes which are involved in regulation of drought response in different stages of plant development. We have then expanded our comparative analysis to species level to identify conserved and species-specific drought-responsive genes in soybean and *Arabidopsis* by comparing our soybean transcriptome datasets and that of drought stressed *Arabidopsis* leaves. Finally, our interest in research on the functions of the *NAC* TF family members and genes involved in hormone metabolism and hormone signaling pathways under drought stress has prompted us to carry out a detailed analysis of the *GmNAC* TF family and the hormone-related gene category. This study ultimately provides excellent candidates for in-depth characterization and future development of improved drought-tolerant transgenic soybeans.

**Table 1:** Confirmation of microarray data by qRT-PCR analysis.

Names	Glyma ID	V6-D/V6-C						R2-D/R2-C					
		qRT-PCR			Soybean whole transcript array			qRT-PCR			Soybean whole transcript array		
		*Fold change*		*p-value*	*Fold change*		*p-value*	*Fold change*		*p-value*	*Fold change*		*p-value*
*GmSGR1*	*Glyma11g02980*	1.3	UP	0.2055	2	UP	0.0182	2.3	UP	0.0162	2.1	UP	0.0092
*GmSGR2*	*Glyma01g42390*	3.3	UP	0.0043	3.3	UP	0.0019	13.5	UP	0.0002	7.0	UP	0.0003
*GmSARK*	*Glyma13g34100*	4.4	DOWN	0.0072	2.2	DOWN	0.0023	1.6	UP	0.1685	1.5	UP	0.1948
*GmCKX01*	*Glyma19g31620*	275.4	DOWN	0.0058	20.8	DOWN	0.0001	2.4	DOWN	0.0110	1.2	DOWN	0.3199
*GmCKX02*	*Glyma03g28910*	416.5	DOWN	0.0004	24.6	DOWN	0.0003	3.4	DOWN	0.0197	3.7	DOWN	0.1326
*GmCKX12*	*Glyma09g35950*	3.3	UP	0.0682	6.5	UP	0.0061	2.6	DOWN	0.0001	2.4	DOWN	0.0009
*GmCKX13*	*Glyma11g20860*	3629.5	DOWN	0.0259	37.4	DOWN	0.0004	25.0	DOWN	0.0000	9.9	DOWN	0.0026
*GmCKX14*	*Glyma12g01390*	18.4	UP	0.0046	9.3	UP	0.0026	2.4	UP	0.0018	2.5	UP	0.0157
*GmCKX15*	*Glyma04g05840*	416.5	DOWN	0.0183	14.4	DOWN	0.0005	5.6	DOWN	0.0022	1.9	DOWN	0.0424
*GmCKX17*	*Glyma17g34330*	6.8	DOWN	0.0000	3.8	DOWN	0.0019	4.3	DOWN	0.0001	2.8	DOWN	0.0002

## Materials and Methods

### Plant Growth, Drought Treatments and Tissue Collections

Soybean plants (cv. Williams 82) were grown in pots (3 plants per 6-liter pot) containing Supermix (Supermix A, Sakata, Japan). Water was given to the pots once a day under greenhouse conditions (continuous 30°C temperature, photoperiod of 12 h/12 h, 80 µmol m^−2^ s^−1^ photon flux density and 50% relative humidity). For the collection of well-watered and drought-stressed V6 leaves, soybean plants at V6 stage (28 days after sowing, containing 7 trifoliates) were withheld from watering to initiate the drought treatment. Water was provided to the well-watered control plants to maintain the volumetric soil moisture content (SMC) at 40–45%. At the sixth day of water withholding (containing 8 trifoliates and beginning bloom), where the SMC was below 5% and the soybean plants contained 7 fully open trifoliates and a half-open 8^th^ trifoliate, soybean leaves were separately collected from each trifoliate leaf. The 3^rd^, 5^th^ and 7^th^ trifoliate leaves were used for determination of the stress severity by measuring leaf relative water content (RWC). The leaf RWC of the stressed plants was approximately 60% of the well-watered plants under our experimental conditions [16]. At the same time, the 4^th^ trifoliate leaves were quickly frozen in liquid nitrogen and stored at −80°C for the isolation of RNA for qRT-PCR or microarray analyses. All of the samples were collected in four biological replicates.

**Figure 1:**
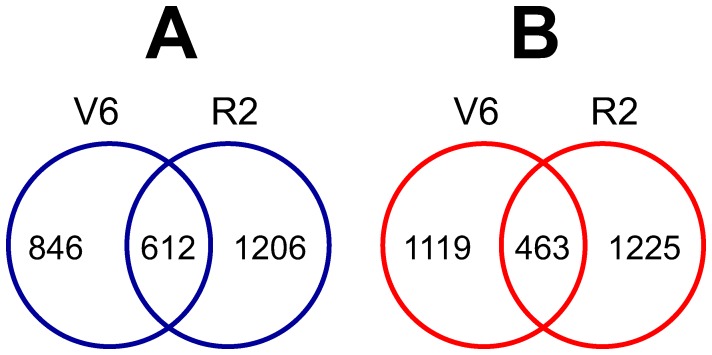
Venn diagram analysis of differentially expressed gene sets of comparision V6-D/V6-C and R2-D/R2-C. (A) Numbers of the overlapping and non-overlapping upregulated genes. (B) Number of the overlapping and non-overlapping downregulated genes.

For the collection of well-watered and drought-treated leaves at the R2 reproductive stage, soybean plants were grown in the pots and drought stress treatment was performed as previously described [16]. The 3^rd^ trifoliate leaves (counting down from the growing shoots) with similar chlorophyll indexes were collected from well-watered (SMC of 30%, leaf RWC = 91±1%) and drought-stressed plants (SMC of 5%, leaf RWC = 32±2%) in three biological replicates for the isolation of RNA for qRT-PCR or microarray analysis.

**Figure 2:**
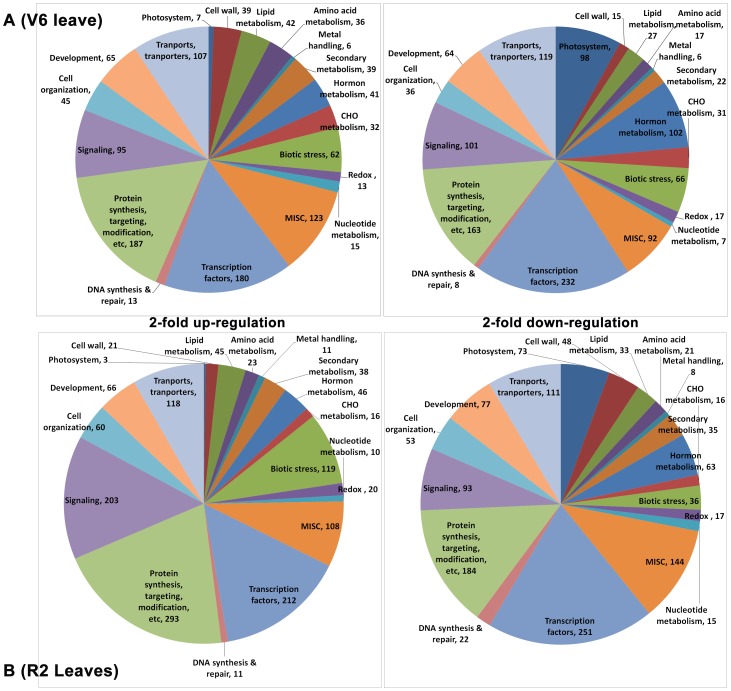
Distribution of up- and down-regulated genes into major biological processes. MapMan was used to classify the genes into the functional categories. Gene numbers are displayed next to the terms.

### RNA Isolation, DNAse Treatment and cDNA Synthesis for qRT-PCR

RNAs were purified using Trizol reagent according to a manufacturer-recommended protocol. DNAse I treatment and cDNA synthesis were performed as previously described [17].

**Table 2:** Differential expression of *GmNAC* genes in different tissues under drought stress.

Probe ID	Glyma ID	Nomenclature^a^	Fold change(V6-D/V6-C)	q-value	Inducibility insoybean seedlings^b^	Inducibility incomparison R2-D/R2-C	Inducibility of*Arabidopsis* ortholog^c^
**Upregulated ** ***NAC*** ** genes**							
11787810	*Glyma02g07700*	*GmNAC006*	2.717	0.042	Shoots↑	Up	Up
11803935	*Glyma02g26480*	*GmNAC011*	4.766	0.012	Shoots↑	Up	Up
11794073	*Glyma02g38710*	*GmNAC012*	2.966	0.018	N/A	Up	Up
11851380	*Glyma04g38560*	*GmNAC018*	4.955	0.020	Shoots↑	Unchanged	Up
11852067	*Glyma04g40450*	*GmNAC021*	2.374	0.015	N/A	Unchanged	Up
11878716	*Glyma06g11970*	*GmNAC035*	5.248	0.042	N/A	Unchanged	Up
11892617	*Glyma06g14290*	*GmNAC036*	4.459	0.013	N/A	Unchanged	Up
11893198	*Glyma06g15990*	*GmNAC038*	44.304	0.010	Shoots↑, roots↑	Up	Unchanged
11880576	*Glyma06g16440*	*GmNAC039*	4.498	0.015	N/A	Unchanged	Up
11884391	*Glyma06g38410*	*GmNAC043*	11.226	0.019	Shoots↑, roots↑	Up	Up
11936212	*Glyma08g41990*	*GmNAC064*	2.162	0.028	N/A	Unchanged	Unchanged
12003799	*Glyma11g07990*	*GmNAC076*	3.962	0.027	N/A	Unchanged	Unchanged
12039219	*Glyma12g22880*	*GmNAC085*	10.982	0.029	Shoots↑	Up	Up
12042020	*Glyma12g35000*	*GmNAC092*	45.245	0.006	Shoots↑, roots↑	Up	Up
12054727	*Glyma13g35550*	*GmNAC101*	27.689	0.011	Shoots↑, roots↑	Up	Unchanged
12087895	*Glyma14g24220*	*GmNAC109*	3.39	0.022	Shoots↑, roots↑	Up	Up
12094383	*Glyma15g08480*	*GmNAC114*	3.013	0.031	N/A	Up	Unchanged
12124443	*Glyma16g04720*	*GmNAC123*	5.548	0.012	N/A	Up	Unchanged
**Downregulated** ***NAC*** **genes**							
11911738	*Glyma07g40140*	*GmNAC053*	−2.129	0.009	N/A	Unchanged	Down
11926243	*Glyma08g08010*	*GmNAC057*	−27.659	0.028	Shoots↑, roots↑	Down	Unchanged
12026921	*Glyma12g09670*	*GmNAC082*	−4.595	0.025	N/A	Unchanged	Up
12054733	*Glyma13g35560*	*GmNAC102*	−7.008	0.005	Shoots↑, roots↑	Unchanged	Unchanged
12142509	*Glyma17g00650*	*GmNAC130*	−3.136	0.017	N/A	Down	Down

Expression of *GmNAC* genes with altered expression in drought-stressed V6 leaves was compared with that of respective *GmNAC* genes in dehydrated shoot or root tissues of 12-d-old soybeen seedlings or that of respective *GmNAC* genes in drought-stressed R2 leaves or that of the best orthologous genes in drought-stressed *Arabidopsis* leaves.

a According to [41].

b Inducibility of *GmNAC* genes in dehydrated shoot and root tissues of 12-d-old soybean seedlings [41]. ↑ arrow indicates upregulation.

c Inducibility of the best *Arabidopsis ANAC* orthologous genes in leaves of 35-d-old *Arabidopsis* seedlings subjected to progressive drought stress [22].

N/A: expression of these *GmNAC* genes was not examined in dehydration-treated root and shoot tissues of soybean seedlings [41].

**Table 3:** Differential expression of *GmNAC* genes in different tissues under drought stress.

Probe ID	Glyma ID	Nomenclature^a^	Fold change(R2-D/R2-C)	q-value	Inducibility in soybean seedlings^b^	Inducibility in comparison V6-D/V6-C	Inducibility of *Arabidopsis* ortholog^c^
**Upregulated ** ***NAC*** ** genes**							
11787810	*Glyma02g07700*	*GmNAC006*	23.254	0.006	Shoots↑	Up	Up
11803935	*Glyma02g26480*	*GmNAC011*	2.085	0.023	Shoots↑	Up	Up
11794073	*Glyma02g38710*	*GmNAC012*	3.286	0.006	N/A	Up	Up
11818170	*Glyma03g35570*	*GmNAC014*	6.364	0.034	N/A	Unchanged	Up
11893198	*Glyma06g15990*	*GmNAC038*	16.077	0.009	Shoots↑, roots↑	Up	Unchanged
11884391	*Glyma06g38410*	*GmNAC043*	11.042	0.012	Shoots↑, roots↑	Up	Up
11884399	*Glyma06g38440*	*GmNAC044*	3.417	0.009	Shoots↑, roots↑	Unchanged	Unchanged
11902484	*Glyma07g05360*	*GmNAC046*	5.088	0.049	N/A	Unchanged	Up
11930140	*Glyma08g18470*	*GmNAC061*	5.525	0.034	Shoots↑	Unchanged	Up
11930485	*Glyma08g19300*	*GmNAC062*	2.056	0.032	Shoots↑, roots↑	Unchanged	Unchanged
12039206	*Glyma12g22790*	*GmNAC084*	4.412	0.017	Shoots↑	Unchanged	Unchanged
12039219	*Glyma12g22880*	*GmNAC085*	26.645	0.010	Shoots↑	Up	Up
12042020	*Glyma12g35000*	*GmNAC092*	17.901	0.010	Shoots↑, roots↑	Up	Up
12054727	*Glyma13g35550*	*GmNAC101*	21.146	0.008	Shoots↑, roots↑	Up	Unchanged
12054733	*Glyma13g35560*	*GmNAC102*	2.509	0.040	Shoots↑, roots↑	Unchanged	Unchanged
12087895	*Glyma14g24220*	*GmNAC109*	3.107	0.019	Shoots↑, roots↑	Up	Up
12094383	*Glyma15g08480*	*GmNAC114*	3.068	0.011	N/A	Up	Unchanged
12112992	*Glyma15g40510*	*GmNAC115*	4.218	0.030	N/A	Unchanged	Up
12114652	*Glyma16g01940*	*GmNAC121*	3.748	0.038	N/A	Unchanged	Up
12124443	*Glyma16g04720*	*GmNAC123*	2.931	0.046	N/A	Up	Unchanged
12120373	*Glyma16g26740*	*GmNAC127*	19.644	0.015	N/A	Unchanged	Up
12181630	*Glyma19g28480*	−d	6.608	0.022	N/A	Unchanged	Up
12181644	*Glyma19g28520*	*GmNAC144*	2.436	0.018	N/A	Unchanged	Unchanged
**Downregulated ** ***NAC*** ** genes**							
11926243	*Glyma08g08010*	*GmNAC057*	−5.768	0.022	Shoots↑, roots↑	Down	Unchanged
11952836	*Glyma08g47520*	*GmNAC065*	−5.854	0.016	N/A	Unchanged	Unchanged
11993366	*Glyma10g20830*	−d	−3.542	0.037	N/A	Unchanged	Unchanged
12142509	*Glyma17g00650*	*GmNAC130*	−2.096	0.048	N/A	Down	Down

Expression of *GmNAC* genes with altered expression in drought-stressed R2 leaves was compared with that of respective *GmNAC* genes in dehydrated shoot or root tissues of 12-d-old soybeen seedlings or that of respective *GmNAC* genes in drought-stressed V6 leaves or that of the best orthologous genes in drought-stressed *Arabidopsis* leaves.

a According to to [41].

b Inducibility of *GmNAC* genes in dehydrated shoot and root tissues of 12-d-old soybean seedlings [41]. ↑ arrow indicates upregulation.

c Inducibility of the best *Arabidopsis ANAC* orthologous genes in leaves of 35-d-old *Arabidopsis* seedlings subjected to progressive drought stress [22].

d These NAC-like proteins with truncated annotated sequence were not included into [41] study.

N/A: expression of these *GmNAC* genes was not examined in dehydration-treated root and shoot tissues of soybean seedlings to [41].

### Microarray Analysis of the Differential Expression in V6 and R2 Leaves Under Normal and Drought Conditions using 61K Affymetrix Microarray

Total RNA was extracted from the trifoliate leaves using Trizol and DNAse I treatment was performed prior to quality assessment of the purified RNA by an Agilent 2100 Bioanalyzer. cDNA synthesis, cRNA amplification and conversion to sense strand cDNAs were performed according to the manufacturer's instructions using the Ambion WT expression kit.

**Figure 3:**
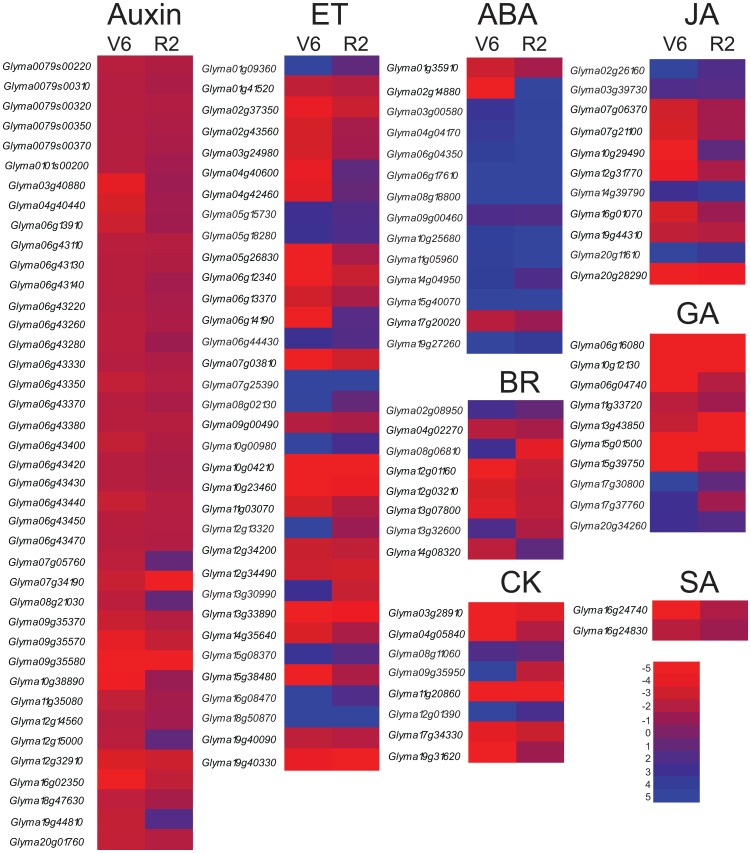
Heatmap analysis of hormone-related genes differentially expressed in soybean V6 and R2 leaves under drought stress. Genes shown are either up-regulated or down-regulated at least by two-fold. Blue and red color gradients indicate an increase or decrease in transcript abundance, respectively. Auxin (IAA, indole-3-acetic acid), ethylene (ET), ABA, jasmonate (JA), giberelline (GA), brassinosteroid (BR), cytokinin (CK) and salicylic acid (SA).

Sense strand cDNAs were then fragmented and end-labeled using Affymetrix GeneChip WT Terminal Labeling Kit according to the manufacturer's instructions. After hybridization, raw data from.CEL files were initially analyzed by Affymetrix Expression Console with library files supplied by Affymetrix. Analyses were performed using the RMA method for gene levels without normalization. The log2-transformed data were exported individually for analysis using GeneSpring software. The data in text format were imported into GeneSpring and normalized using a 75-percentile threshold. A Student’s *t*-test was used to analyze the statistical significance of the same gene in different conditions. Benjamini and Hochberg False Discovery Rate were used to calculate the corrected p-values (q-values). All of these statistical tests were performed using GeneSpring Ver. 11.

Annotation of the microarray data was performed using MAPMAN map file based on Glyma ver. 1.09. Briefly, the map file was first modified to replace the transcript IDs by the gene IDs. All of the redundant IDs were then deleted to obtain one unique record per gene ID. Finally, various functions in MS Excel were used to match the Glyma IDs, which have annotations with that of the microarray data. All Glyma IDs that were annotated in the Mapman file but lacked an original description were further excluded. Thus, a database of 41059 genes with microarray data was annotated [18].

### qRT-PCR and Statistical Analysis of the Data

Specific primer pairs for qRT-PCR were designed for *GmSGR1* (F: 5′-ACGCATCTAAACACTCCTCCGAACT-3′ and R: 5′-GTGTGGGGGAGCTATAGGTTTTGCT-3′), *GmSGR2* (F: 5′- GGCCAAGGAAGAGAGTGAGCAAG-3′and R: 5′- TGGGCTTAACGTCAGCGGTGG-3′) and *GmSARK* (F: 5′-GCCAATGGCACCGTCTGCCA-3′ and R: 5′-CCGAGGGAGAGTGCCAGGGA-3′) as previously described [17]. The *CYP2* gene was used as a reference gene in the expression profiling of soybean genes [19]. qRT-PCR reactions and data analyses were performed according to previously published methods [17].

## Results and Discussion

### Transcriptome Analysis of Soybean V6 and R2 Leaves under Normal and Drought Conditions using Microarray

With the availability of the whole genomic sequence of soybean and the Glyma1 annotation, which predicted 46430 protein-coding genes with high confidence out of approximately 66000 putative genes [15], Affymetrix has designed the newest version of the Soybean Array GeneChip covering all of the soybean putative genes. To gain a comprehensive overview of the transcriptome-wide regulation in soybean leaves under drought stress during the critical period spanning from late V6 stage toward the end of R2 stage, we performed microarray analyses to compare the transcriptome changes of drought-stressed V6 leaves vs. well-watered V6 leaves (comparison V6-D/V6-C) and drought-stressed R2 leaves vs. well-watered R2 leaves (comparison R2-D/R2-C) using this 66 K Affymetrix Soybean Array GeneChip. Our analysis indicated that this array version contains 66555 probes. Within this array, 66195 probes matched with the respective gene IDs used in the Glyma1 model, corresponding to 99.46% of the total probes (Tables S1, S2, Gene Expression Omnibus accession number: GSE40627). With reference to the annotation of the Glyma1 version [15], of all the genes identified on the 66 K Affymetrix Soybean Array GeneChip, 46093 genes could be annotated (Tables S3A, S4A). Among the genes that could be annotated, 41059 genes could be assigned with a known function, and these genes were used in further analyses (Tables S3B, S4B). Using the criteria of a ratio change > = 2 and a q-value<0.05, 1458 and 1582 genes were found to be upregulated and downregulated, respectively, in drought-stressed V6 leaves (Tables S3C, S3D). When the same criteria were applied for comparison R2-D/R2-C, we were able to detect 1818 upregulated and 1688 downregulated genes. For verification of our microarray data, first we selected three well-known senescence-inducible genes, two soybean stay-green *GmSGR1* and *GmSGR2* genes [20] and a senescence-associated receptor-like kinase *GmSARK* gene [21], and assessed their expression by qRT-PCR. Next, as another mean of verification we compared expression profiles of 7 *GmCKX* genes that were obtained by systematic expression analysis of cytokinin (CK)-metabolic genes using qRT-PCR [16] with their respective expression data obtained by our microarray analysis. Results shown in Table 1 indicated good accordance between the microarray and qRT-PCR data, suggesting that the newly designed 66 K Affymetrix Soybean Array GeneChip is useful for genome-wide expression profiling of soybean genes in leaf tissue, and perhaps in other tissues as well, under drought stress.

Both the up- and downregulated gene sets identified in two comparisons V6-D/V6-C and R2-D/R2-C were subjected to a Venn diagram analysis to identify overlapping genes between the two comparisons. The altered gene expression profile of the drought-treated V6 leaves was significantly different from that of the drought-treated R2 leaves (Figure 1). Specifically, when the up- and downregulated gene sets identified in comparison V6-D/V6-C were compared with the corresponding gene sets from comparison R2-D/R2-C, overlap was observed for only 41.98% and 29.27% of the up- and downregulated gene sets of comparison V6-D/V6-C, respectively. This result suggests that a significant number of genes respond to drought stress in stage-specific manner. Alternatively, the different stress effect might play a role. Under our experimental conditions, although the drought stress was maintained until the SMC reached 5% in both cases, the RWCs were approximately 98±1% and 55±2% for well-watered and drought-treated V6 leaves, respectively, while the respective values were about 91±1% and 32±2% for well-watered and drought-treated R2 leaves [16].

### Functional Classification of the Differentially Expressed Drought-responsive Gene Sets

Drought stress results in dramatic losses of the yield of various crops by adversely affecting their growth and physiology. As a result, in response to drought stress plants have developed strategies to increase their defense against water deficit conditions. A comparative expression analysis of the up- and downregulated gene sets identified in drought-stressed soybean V6 and R2 leaves against the transcriptome of drought stressed leaves of 35-d-old *Arabidopsis* plants [22] has demonstrated that many soybean and *Arabidopsis* orthologous genes are either drought-inducible or drought-repressible in a similar manner, suggesting that the two species may share common mechanisms for drought stress responses (Tables S3C, S3D, S4C, S4D). On the other hand, a small number of *Arabidopsis* orthologs display differential responses to drought, indicating that there is also species-specific drought response (Tables S3C, S3D, S4C, S4D). These results together suggest that in response to drought stress plants activate both common and species-specific mechanisms to survive water stress conditions.

In the next step, to further classify the drought-responsive genes into various biological categories, MapMan was used to visualize the soybean gene expression data for various biological processes and to assign up- and downregulated genes to biological process categories in a systematic manner. This analysis will allow us to obtain an overview on the biological functions of the differentially expressed genes identified in the two comparisons. As shown in Figure 2, for both two comparisons the up- and downregulated gene sets were grouped into the 19 most abundant categories in similar proportions, except for the “Signaling” category, in which the number of the upregulated genes identified in R2-D/R2-C comparison was more than double comparing with that of V6-D/V6-C comparison.

A closer look at the drought-induced gene sets classified the upregulated genes identified in comparisons V6-D/V6-C and R2-D/R2-C into regulatory and functional categories. For the regulatory category, many upregulated genes were grouped into TF, signaling and protein modification groups (Tables S3C, S3D, S4C, S4D). In the TF group, many soybean genes encoding TFs with high homology to the well-known drought/abscisic acid (ABA)-inducible TFs, such as RD26/ANAC072 and ATAF1/ANAC002 of the NAC family (see detailed analysis of the NAC TF family below) and AREB1 of the bZIP family [23]–[27], were upregulated in both two comparisons (Tables S3C, S4C). Heat shock TF and heat shock protein encoding genes were also found among the genes with increased transcript abundance. All of these types of TFs are known to function in plant adaptation to various stresses, including drought [2], [3], [5]–[9], [28], [29]. Interestingly, the soybean genes encoding soybean othologs of the *Arabidopsis* DREB1A and DREB1D of the AP2_EREBP family and ZAT10/STZ of the C2H2_Zn family were induced in drought-stressed V6 leaves but not in drought-stressed R2 leaves, suggesting that these TFs may be involved in regulation of drought response during vegetative growth (V6) rather than reproductive growth (R2); a phenomenon indicating developmental stage-specific function of TFs. For the signaling and protein modification groups, we identified many drought-inducible genes encoding kinases, such as CIPKs and MAP kinases, PP2C proteins and hormone-signaling related proteins (see detailed analysis below), which were reported to be involved in the regulation of the drought response [9], [30], [31]. The functional category contained many upregulated genes encoding LEA proteins, ABA metabolism-related proteins, osmoprotectant biosynthesis-related proteins, transporters and detoxification enzymes (Tables S3C, S3D).

With regard to the downregulated gene sets, the major difference found between the up- and downregulated gene sets was that many photosynthesis-related genes were down-regulated under drought stress in both two comparisons (Figure 2). This finding is consistent with previously published results as photosynthesis is negatively affected by various stresses, including drought [12], [32]–[34]. The downregulation of photosynthesis-related genes, which contribute to, at least in part, growth retardation under drought stress, may serve as an adaptive mechanism for plant survival.

### Differential Expression of the *NAC* TF Family Members in Drought-stressed V6, R2 Leaves and Dehydrated Shoots and Roots of Young Soybean Seedlings

As previously discussed, drought stress has altered expression of many TF encoding genes in the soybean V6 and R2 leaves which belong to different TF families. Among the major TF families, the NAC TF family has been shown to provide many useful candidate genes for genetic engineering of improved drought-tolerant plants [2], [28], [35], [36]. The first evidence demonstrating the functions of NAC TFs in the improvement of drought tolerance in plants was reported in *Arabidopsis* by the overexpression of the *ANAC019*, *ANAC055* and *ANAC072* genes [23], [37]. Following this work, a number of studies on abiotic stress-related functions of NAC TFs in various plant species, including important crops such as rice and wheat, have been reported [35], [36], [38], even in field trials [39], [40].

Given the biotechnological potentials of the NAC family, in this section we aimed to analyze in detail the drought-responsive expression profiles of NAC TF family in drought-stressed V6 and R2 leaves. Among 41059 genes that could be assigned with a known function, 175 putatively annotated *GmNAC* genes were identified (Tables S3B, S4B). Out of these *GmNAC* genes, a total of 18 and 4 genes were found to be upregulated and downregulated by more than two-fold (q-value<0.05) in the soybean V6 leaves by drought stress (Table 2), while using the same criteria 23 and 4 genes were upregulated and downregulated in drought-treated R2 leaves (Table 3). Previously, expression analysis of 38 *GmNAC* genes in dehydrated shoots and roots of 12-d-old soybean seedlings using qRT-PCR has found 29 and 6 *GmNAC* genes upregulated and downregulated, respectively, in dehydrated shoot and/or root tissues [41]. Comparison of the differential expression of the *GmNAC* genes in drought-stressed V6 and R2 leaves and dehydrated shoot and root tissues of 12-d-old soybean seedlings revealed that all the *GmNAC* genes upregulated in the drought-stressed V6 and R2 leaves were also upregulated in the dehydrated shoot and/or root tissues of 12-d-old soybean seedlings if their expression was examined in these tissues (Tables 2, 3). In addition, more than half of the *GmNAC* genes induced in drought-stressed V6 leaves were also upregulated in drought-stressed R2 leaves and vice versa (Tables 2, 3). Furthermore, a comparative analysis against the expression of the *Arabidopsis NAC* orthologous genes in leaves of 35-d-old *Arabidopsis* plants, which were subjected to a soil drought treatment [22], indicated that the majority of the soybean and *Arabidopsis NAC* orthologous genes were drought-responsive in a similar manner, suggesting the existence of a relatively well-conserved drought response in the leaves of the two dicotic species at similar developmental stage. This comparative analysis may help us select drought-responsive soybean *GmNAC* genes with more confidence for further studies and genetic engineering. On the other hand, among 5 and 4 *GmNAC* genes significantly downregulated in the drought-stressed V6 and R2 leaves, respectively, two genes, *GmNAC057* and *GmNAC102*, were examined transcriptionally in the dehydrated shoot and root tissues of 12-d-old soybean seedlings. Interestingly, unlike in the drought-stressed V6 or R2 leaves the expression of these two genes was upregulated in the dehydrated root tissue and unchanged in the dehydrated shoot tissue of young soybean seedlings in comparison with the untreated controls (Tables 2, 3). These results together suggest that the dynamics of drought -responsive expression of the *NAC* genes in soybean is complex. Stresses may trigger different stress-responsive gene expression in different tissues at the same developmental stage or in the same tissue at different developmental stages. This characteristic of the *GmNAC* gene family, and perhaps other gene families, will enable us to perform genetic engineering in an organ-specific and/or developmental stage-specific manner.

### Differential Expression of Hormone-related Genes in V6 and R2 Leaves under Drought Stress

It is well established that various plant hormones, such as ABA, cytokinin (CK) and brassinosteroid (BR), and their respective hormone pathways are involved in regulation of drought stress responses [42], [43]. Conversely, stresses are known to influence the expression of hormone-related genes, including those involved in hormone metabolisms and hormone signaling pathways, leading to changes in hormone homeostasis, redistribution and signaling [12], [42], [44]–[46]. To have an overview on the expression profiles of hormone-related genes in the V6 and R2 leaves under drought stress, MapMan was used to visualize the gene expression data of the annotated hormone-related genes which were generated by our microarray analysis. In our study, we examined the expression levels of both biosynthetic and signaling genes related to auxin (IAA, indole-3-acetic acid), ethylene (ET), ABA, jasmonic acid (JA), giberelline (GA), brassinosteroid (BR), CK and salicylic acid (SA).Within the Glyma 1 annotation, we detected hormone-related genes which have significant change in expression levels by at least two-fold in drought-stressed V6 leaves (Table S5). The expression of these genes in drought-stressed R2 leaves was also extracted from comparison R2-D/R2-C for comparative analysis (Figure 3, Table S5). Auxin-related gene family showed the highest number with 40 members having differential expression. All the auxin-related genes identified were downregulated in the drought-stressed V6 leaves, and the majority of these genes showed reduced expression in the drought-stressed R2 leaves as well. This finding is in agreement with the results reported previously, in which the authors reported that almost all the auxin-related genes were downregulated in *Arabidopsis* whole plants [12] and *Sorghum bicolor* leaves [44] under drought stress. Among the genes related to the hormones analyzed, ET-related genes formed the second major group with 34 genes displaying altered expression profiles in drought-stressed R2 and/or V6 leaves. A significant proportion of ET-related genes showed the same expression patterns in both V6 and R2 leaves, while only a few genes exhibited opposite expression profiles in the two leaves of different developmental stages under drought stress. ABA-related genes made the third biggest group with 14 members out of which 11 and 12 genes were remarkably induced in V6 and R2 leaves, respectively, by drought stress. Recently, BRs and CKs were shown to be involved in regulation of plant responses to drought stress, and genetic engineering of the homeostasis of these two hormones at biosynthesis or signaling levels enhanced tolerance to various stresses, including drought [42], [43], [45], [47]–[50]. Our microarray analysis identified 8 BR-related and 8 CK-related genes showing altered expression in drought-stressed R2 and/or V6 leaves. The majority of the *GmCKX* genes were downregulated in both two types of soybean leaves under drought stress which is in consistence with the result reported previously in *Arabidopsis* [45]. Similar to BR and CK, GA plays an important role in plant responses to both biotic and abiotic stresses [42], [51]–[53]. Ten GA-related genes were recorded with altered expression in our study, and the majority of these genes exhibited downregulated expression in both the drought-stressed leaves. SA and JA are known as hormones regulating mainly biotic stress responses. Eleven JA-related and 2 SA-related genes were found to have differential expression in R2 and/or V6 leaves under imposed drought conditions, providing evidence that JA and SA may be involved in regulation of drought responses as well.

### Conclusions

Microarray analysis is a comprehensive and high-throughput approach used to screen candidate genes and predict gene function. The availability of the 66 K soybean Array GeneChip has allowed us to acquire large-scale transcriptional changes at a genome-wide level and has identified genes involved in the drought response in soybean. Furthermore, huge amounts of transcriptomic data obtained from microarray analyses of various plant species under drought stress have enabled us to carry out comparisons of drought-responsive expression profiles of different plant species. Our data demonstrate that drought stress triggers both conserved and species-specific responses to water deficit conditions; a result that encourages us to “translate” basic scientific discoveries achieved using model plants into economically important crops and allows us to dissect the species-specific regulatory mechanisms. Additionally, within species several genes may be involved in regulation of drought responses in a specific manner, depending on developmental stages and/or stress effect. Overall, this study provides a basic foundation for further analyses of functions of drought-responsive candidate genes which ultimately lead to development of drought-tolerant soybean cultivars.

Accession number to microarray data deposited at Gene Expression Omnibus database: GSE40627. During review of the paper, data can be freely accessed at http://www.ncbi.nlm.nih.gov/geo/query/acc.cgi?token=flovxwkayiisora&acc=GSE40627.

## Supporting Information

Table S1 **Expression data of annotated soybean genes in V6 leaves under drought stress.** Data received from microarray analysis of drought-stressed V6 leaves using the 66 K Affymetrix Soybean Array GeneChip.
(XLSX)

Table S2 **Expression data of annotated soybean genes in R2 leaves under drought stress.** Data received from microarray analysis of drought-stressed R2 leaves using the 66 K Affymetrix Soybean Array GeneChip.
(XLSX)

Table S3 **Differential expression data of soybean genes in V6 leaves under drought stress.** (A) Numbers of genes on the 66 K Affymetrix Soybean Array GeneChip that could be annotated. (B) Microarray analysis of genes that are annotated with a function. (C) List of upregulated genes which have an annotated function. (D) List of downregulated genes which have an annotated function.
(XLSX)

Table S4 **Differential expression data of soybean genes in R2 leaves under drought stress.** (A) Numbers of genes on the 66 K Affymetrix Soybean Array GeneChip that could be annotated. (B) Microarray analysis of genes that are annotated with a function. (C) List of upregulated genes which have an annotated function. (D) List of downregulated genes which have an annotated function.
(XLSX)

Table S5 **Differential expression of hormone-related genes in soybeanV6 and R2 leaves under drought stress.**
(XLS)
